# The association of lipoprotein(a) and intraplaque neovascularization in patients with carotid stenosis: a retrospective study

**DOI:** 10.1186/s12872-021-02038-x

**Published:** 2021-06-09

**Authors:** Shuang Xia, Weida Qiu, Anping Cai, Bo Kong, Lan Xu, Zejia Wu, Liwen Li

**Affiliations:** 1 The Second School of Clinical Medicine, Southern Medical University, Guangzhou, 510515 China; 2 Department of Cardiology, Guangdong Cardiovascular Institute, Guangdong Provincial People’s Hospital, Guangdong Academy of Medical Sciences, 106 of Zhongshan 2nd Road, Guangzhou, 510100 Guangdong China

**Keywords:** Contrast-enhanced ultrasound, Lipoprotein(a), Intraplaque neovascularization, Carotid stenosis, Cardiovascular events

## Abstract

**Background:**

Lipoprotein(a) is genetically determined and increasingly recognized as a major risk factor for arteriosclerotic cardiovascular disease. We examined whether plasma lipoprotein(a) concentrations were associated with intraplaque neovascularization (IPN) grade in patients with carotid stenosis and in terms of increasing plaque susceptibility to haemorrhage and rupture.

**Methods:**

We included 85 patients diagnosed with carotid stenosis as confirmed using carotid ultrasound who were treated at Guangdong General Hospital. Baseline data, including demographics, comorbid conditions and carotid ultrasonography, were recorded. The IPN grade was determined using contrast-enhanced ultrasound through the movement of the microbubbles. Univariate and multivariate binary logistic regression analyses were used to evaluate the association between lipoprotein(a) and IPN grade, with stepwise adjustment for covariates including age, sex, comorbid conditions and statin therapy (model 1), total cholesterol, triglyceride, low-density lipoprotein cholesterol calculated by Friedwald's formula, high-density lipoprotein cholesterol, apolipoprotein A and apolipoprotein B (model 2), maximum plaque thickness and total carotid maximum plaque thickness, degree of carotid stenosis and internal carotid artery (ICA) occlusion (model 3).

**Results:**

Lipoprotein(a) was a significant predictor of higher IPN grade in binary logistic regression before adjusting for other risk factors (odds ratio [OR] 1.238, 95% confidence interval [CI] (1.020, 1.503), *P* = 0.031). After adjusting for other risk factors, lipoprotein(a) still remained statistically significant in predicting IPN grade in all model. (Model 1: OR 1.333, 95% CI 1.074, 1.655, *P* = 0.009; Model 2: OR 1.321, 95% CI 1.059, 1.648, *P* = 0.014; Model 3: OR 1.305, 95% CI 1.045, 1.628, *P* = 0.019). Lp(a) ≥ 300 mg/L is also significantly related to IPN compare to < 300 mg/L (OR 2.828, 95% CI 1.055, 7.580, *P* = 0.039) as well as in model 1, while in model 2 and model 3 there are not significant difference.

**Conclusions:**

Plasma lipoprotein(a) concentrations were found to be independently associated with higher IPN grade in patients with carotid stenosis. Lowering plasma lipoprotein(a) levels may result in plaque stabilization by avoiding IPN formation.

## Background

Due to recent medical developments, many carotid artery wall imaging methods are available to diagnose and assess carotid stenosis, such as ultrasound, computed tomography, magnetic resonance imaging, and even catheter-based angiography [1]. However, few methods can detect intraplaque neovascularization (IPN), which increases the susceptibility to haemorrhage and rupture of the plaque [2] that are also expensive and inconvenient to deal with in terms of repeated examinations. Contrast-enhanced ultrasound (CEUS) can reliably detect IPN [3] through identifying the movement of the contrast microbubbles within the plaque [4].

Lipoprotein(a) (Lp(a)), formed from an apolipoprotein (apo) B-100 covalently linked to apo(a), is a low-density lipoprotein (LDL)-like protein which is genetically determined and increasingly recognised as a major risk factor for ASCVD [5]. Previous studies have demonstrated a strong and independent association between Lp(a) and carotid artery disease [6] and Lp(a) independently predict carotid atherosclerosis progression [7]. However, for a great degree of homology between apo(a) and plasminogen [8], lots of studies considered the Lp(a) fragments showed an anti-angiogenesis role in vitro [9, 10]. On the contrast, there are also a great deal of conflicting reports postulated Lp(a) induced angiogenesis [11] while others demonstrated a neutral effect on angiogenesis [12], engendering considerable controversy. So, whether Lp(a) is associate with IPN across the plaque, affecting its stability, is worth pondering.

This research aimed to study the association between IPN and plasma Lp(a) concentrations and analyse the role of Lp(a) in relation to the carotid artery in patients with carotid stenosis using CEUS.

## Methods

### Study design and population

This retrospective study enrolled 85 consecutive patients who were diagnosed with carotid stenosis using carotid artery ultrasonography (US) in Guangdong General Hospital, China, from January 2017 to January 2020. The inclusion criteria were as follows: (1) carotid stenosis confirmed using carotid artery ultrasonography, (2) absence of clinical contraindications for CEUS, and (3) ≥ 18 years of age. The exclusion criteria were: (1) having undergone previous carotid endarterectomy, (2) no outcome data concerning Lp(a), and (3) declining to be involved initially or in follow-up. All patients underwent CEUS after being diagnosed with carotid stenosis using carotid artery ultrasonography. Clinical histories, along with demographic and clinical data, were recorded for all patients at admission. All patients provided written informed consent. This retrospective investigation was approved by the local institutional review board as well as performed in accordance with the Declaration of Helsinki.

### Laboratory Measurements

All fasting venous blood samples were collected during hospital admission before undergoing CEUS. Serum Lp(a) levels were measured through a murine monoclonal antibody (E022-1-1, Bioroyee, Beijing, China) involving latex turbidimetric method. Cholesterol (TC), triglyceride (TG), low-density lipoprotein cholesterol (LDL-C), high-density lipoprotein cholesterol (HDL-C), apolipoprotein(apo) A and apo B levels were determined by chemiluminescence method using an auto-analyser. Friedwald's formula (TC -HDL-C-TG/2.2) was used to calculate LDL-C^(F)^.

### US examinations of the carotid artery

Carotid US examinations were performed in all participants at screened visit. Focal structures protruding into carotid lumen with a height > 1.5 mm or 50% intima-media thickness was defined as atherosclerotic plaques [13]. Maximun vertical distance from the top of plaque to adventitia interface of lumen was measured as maximum internal carotid artery (ICA) plaque thickness. Total maximum plaque thickness was the cumulative total by bilateral maximum ICA plaques thickness. The degree of ICA stenosis was divided into 4 groups: mild, < 50%; moderate, 50–69%; severe, 70–99% [14]; and occlusion, without signal of blood flow.

### CEUS examinations of the carotid artery

Carotid CEUS examinations were performed by a researcher who is blinded to the patients’ histories and characteristics, using a GE Vivid E95 or Philips IU elite diasonograph contrast model and a high-frequency superficial probe. CEUS was performed with an ultrasound contrast agent, SonoVue. An initial bolus injection of 1.6 mL of contrast agent was quickly administered into the median cubital vein in 2–3 s, immediately followed by 3 mL of 0.9% normal saline solution at the same speed. Ultrasound cine-loops were then recorded over 15–30 s. The images at 3 s before and 5 min after contrast agent was introduced into the carotid artery lumen were stored for real-time dynamic analysis. IPN grade was determined using CEUS grade as follows: 0, no visible microbubbles in the plaque; 1, minimal microbubbles restricted to adventitial side or shoulder of the plaque; or 2, microbubbles spread all over the plaque [15]. We stratified participants into one of two groups based on their CEUS grade, that is, a CEUS grade on both sides that added up to greater than or equal to 2 was used to define an IPN group whereas a CEUS grade on both sides that added up to fewer than 2 was used to define a no IPN group.

### Statistical analysis

All descriptive data consistent with normal distribution are expressed as mean value ± standard deviation, with the rest expressed as median (interquartile range). Discrete data are presented as frequencies and percentages. A Student’s t-test was used to evaluate continuous variables showing a normal distribution and a Mann–Whitney U-test was used to evaluate variables that show a skewed distribution, while categorial variables were performed using Fisher’s exact test. Differences in baseline characteristics were examined between IPN group versus No IPN group (Table 1) and Lp(a) ≥ 300 mg/L vs < 300 mg/L(Table 2). Spearman’s correlation analysis was further performed to analyse the relationship between Lp(a) and maximum ICA plaque thickness, total ICA maximum plaque thickness, degree of carotid stenosis and ICA occlusion, respectively. Univariate binary logistic regression analyses were used to determine the association between Lp(a) and IPN grade (IPN group vs No IPN group). OR and 95% CI was reported. Multivariate binary logistic regression analyses were then performed to evaluate the association between Lp(a) concentration and IPN grade (IPN group vs No IPN group), with stepwise adjustment for covariates including age, sex and comorbid conditions (model 1), total cholesterol, triglyceride, low-density lipoprotein cholesterol calculated by Friedwald's formula, high-density lipoprotein cholesterol, apolipoprotein A and apolipoprotein B (model 2), maximum ICA plaque thickness and total ICA maximum plaque thickness, degree of carotid stenosis and ICA occlusion (model 3). Meanwhile, participants were divided into two groups according to Lp(a) concentration (≥ 300 mg/L and < 300 mg/L). Univariate and multivariate binary logistic regression analyses were then performed to evaluate the association with IPN grade, adjusting for model 1, model 2 and model 3. All analyses were performed with SPSS version 22.0 for Windows, and a two-sided *P* value of less than 0.05 was considered to indicate statistical significance.

**Table 1:** Baseline characteristics comparisons between IPN group and No IPN group of the 85 participants

Variables	Overall (n = 85)	IPN group (n = 36)	No IPN group (n = 49)	*P* value
*General conditions and Comorbid conditions*				
Age (year)	68.48 ± 8.78	70.17 ± 9.06	67.24 ± 8.45	0.130
Male sex, n (%)	63 (74.1%)	27 (75%)	36 (73.5%)	0.874
Hypertension, n (%)	60 (70.6%)	27 (75%)	33 (67.3%)	0.444
Diabetes mellitus, n (%)	26 (30.6%)	14 (38.9%)	12 (24.5%)	0.155
Cerebral infraction, n (%)	21 (24.7%)	7 (19.4%)	14 (28.6%)	0.335
Coronary heart disease, n (%)	38 (44.7%)	17 (47.2%)	21 (42.9%)	0.689
Smoking history, n (%)	20 (23.5%)	6 (16.7%)	14 (28.6%)	0.201
Statin therapy, n (%)	79 (92.9%)	33 (91.7%)	46 (93.9%)	0.695
*Serum lipid*				
Lp(a) (mg/L) *	184 (96.5–370)	260 (92–427.75)	173 (100–258.5)	0.018
TC (mmol/L)	4.49 ± 1.21	4.44 ± 1.10	4.53 ± 1.29	0.732
TG (mmol/L) *	1.34 (1.04–1.99)	1.44 (1.08–2.26)	1.23 (0.87–1.66)	0.223
LDL-C (mmol/L)	2.92 ± 0.91	2.88 ± 0.81	2.95 ± 0.99	0.729
LDL-C^(F)^ (mmol/L)	2.13 ± 0.75	2.07 ± 0.64	2.17 ± 0.82	0.537
HDL-C (mmol/L) *	0.99 (0.87–1.16)	0.98 (0.83–1.16)	1.02 (0.88–1.15)	0.778
Apo(a) (mmol/L)*	1.16 (1.01–1.28)	1.13 (1.00–1.25)	1.16 (1.02–1.30)	0.593
Apo(b) (mmol/L)*	0.79 (0.64–1.01)	0.77 (0.63–0.94)	0.80 (0.64–1.04)	0.536
*Carotid plaque*				
Maximum ICA plaque thickness (mm)	3.89 ± 1.14	4.27 ± 0.90	3.61 ± 1.22	0.008
Total maximum ICA plaque thickness (mm)	6.59 ± 2.12	7.28 ± 1.84	6.08 ± 2.19	0.009
*Degree of carotid stenosis*				
Mild, n (%)	27 (31.8%)	11 (30.6%)	16 (32.7%)	1.000
Moderate, n (%)	14 (16.5%)	6 (16.7%)	8 (16.3%)	1.000
Severe, n (%)	28 (32.9%)	14 (38.9%)	14 (28.6%)	0.356
Occlusion, n (%)	16 (18.8%)	5 (13.9%)	11 (22.4%)	0.405
*Reasons for referral*				
Cardiovascular ischemia-related, n (%)	44 (51.8%)	22 (66.1%)	22 (44.9%)	0.189
Cerebral ischemia-related, n (%)	30 (35.3%)	11 (30.6%)	19 (38.8%)	0.496
Asymptomatic, n (%)	7 (8.2%)	2 (5.6%)	5 (10.2%)	0.694
Other	4 (4.7%)	1 (2.8%)	3 (6.1%)	0.634

LDL-C^(F)^ = TC-HDL-C-TG/2.2

IPN, intraplaque neovascularization; Lp(a), Lipoprotein(a); TC, total cholesterol; TG, triglyceride; LDL-C, low-density lipoprotein cholesterol; LDL-C^(F)^, Friedwald's formula adjust low-density lipoprotein cholesterol; HDL-C, high-density lipoprotein cholesterol; Apo, apolipoprotein; ICA, Internal carotid artery

^*^Presented as median (interquartile range)

**Table 2:** Baseline characteristics comparisons between Lp(a) < 300 mg/L and Lp(a) ≥ 300 mg/L of the 85 participants

Variables	Overall (n = 85)	Lp(a) < 300 mg/L (n = 62)	Lp(a) ≥ 300 mg/L (n = 23)	*P* value
*General conditions and Comorbid conditions*				
Age (year)	68.48 ± 8.78	68.66 ± 9.00	68.00 ± 8.35	0.760
Male sex, n (%)	63 (74.1%)	45 (72.6%)	18 (78.3%)	0.782
Hypertension, n (%)	60 (70.6%)	45 (72.6%)	15 (65.3%)	0.594
Diabetes mellitus, n (%)	26 (30.6%)	21 (33.9%)	5 (21.7%)	0.427
Cerebral infraction, n (%)	21 (24.7%)	15 (24.2%)	6 (26.1%)	1.000
Coronary heart disease, n (%)	38 (44.7%)	24 (38.7%)	14 (60.9%)	0.087
Smoking history, n (%)	20 (23.5%)	14 (22.6%)	6 (26.1%)	0.777
Statin use, n (%)	79 (92.9%)	58 (91.3%)	21 (91.3%)	0.660
*Serum lipid*				
Lp(a) (mg/L) *	184 (96.5–370)	129 (81–209.25)	473 (390–822)	< 0.0001
TC (mmol/L)	4.49 ± 1.21	4.50 ± 1.31	4.48 ± 0.90	0.935
TG (mmol/L) *	1.34 (1.04–1.99)	1.43 (1.04–2.27)	1.27 (0.91–1.47)	0.016
LDL-C (mmol/L)	2.92 ± 0.91	2.93 ± 0.98	2.89 ± 0.70	0.818
LDL-C^(F)^ (mmol/L)	2.13 ± 0.75	2.20 ± 0.80	1.93 ± 0.53	0.088
HDL-C (mmol/L) *	0.99 (0.87–1.16)	0.94 (0.82–1.09)	1.11 (1.01–1.32)	0.005
Apo(a) (mmol/L) *	1.16 (1.01–1.28)	1.10 (0.97–1.30)	1.19 (1.12–1.26)	0.060
Apo(b) (mmol/L) *	0.79 (0.64–1.01)	0.78 (0.62–1.02)	0.80 (0.70–1.03)	0.602
*Carotid plaque*				
Maximum ICA plaque thickness (mm)	3.89 ± 1.14	3.82 ± 1.10	4.03 ± 1.27	0.501
Total maximum ICA plaque thickness (mm)	6.59 ± 2.12	6.49 ± 2.14	6.84 ± 2.10	0.502
*Degree of carotid stenosis*				
Mild, n (%)	27 (31.8%)	21 (33.9%)	6 (26.1%)	0.604
Moderate, n (%)	14 (16.5%)	11 (17.7%)	3 (13.0%)	0.749
Severe, n (%)	28 (32.9%)	16 (25.8%)	12 (52.2%)	0.036
Occlusion, n (%)	16 (18.8%)	14 (22.6%)	2 (8.7%)	0.215
*Reasons for referral, n (%)*				
Cardiovascular ischemia-related, n (%)	44 (51.8%)	33 (53.2%)	11 (47.8%)	0.808
Cerebral ischemia-related, n (%)	30 (35.3%)	18 (29.0%)	12 (52.2%)	0.073
Asymptomatic, n (%)	7 (8.2%)	7 (11.3%)	0 (0.0%)	0.091
Other	4 (4.7%)	4 (6.5%)	0 (0.0%)	0.296

LDL-C(F) = TC-HDL-C-TG/2.2

Lp(a), Lipoprotein(a); TC, cholesterol; TG, triglyceride; LDL-C, low-density lipoprotein cholesterol; LDL-C^(F)^, Friedwald's formula adjust low-density lipoprotein cholesterol; HDL-C, high-density lipoprotein cholesterol; Apo, apolipoprotein; ICA, Internal carotid artery

^*^Presented as median (interquartile range)

## Results

### Participant characteristics

The characteristics of the participants are shown in Table 1 (IPN group vs No IPN group) and Table 2 (Lp(a) ≥ 300 mg/L vs < 300 mg/L). All 85 consecutive patients, that we had complete data for the binary logistic regression analysis, were seen in Guangdong General Hospital from January 2017 to January 2020. The mean age of the population was 68.48 ± 8.78 years. More than 70% of the participants were male, with 92.9% of the participants receiving statin therapy. Approximately 20% were smokers, with a similar proportion suffering from diabetes mellitus and cerebral infarction. Slightly fewer than half of the participants had coronary heart disease, and 70% had hypertension. Half of the patients (51.8%) admitted for cardiovascular ischemia-related reasons, including the symptom of chest pain or shortness of breath. While other 35.3% patients admitted for cerebral ischemia-related reasons including dizzy, blurred vision or unilateral limb weakness and 8.2% patients suffered from asymptomatic carotid stenosis. The IPN group tended to have greater maximum ICA plaque thickness and total maximum ICA plaque thickness compared to No IPN group, while compared to < 300 mg/L group, it was not significant in the Lp(a) ≥ 300 mg/L group. And Lp(a) ≥ 300 mg/L group have lower TG and higher HDL-C level than the Lp(a) < 300 mg/L group.

Spearman’s correlation analysis was performed to evaluate whether Lp(a) was associate with ICA plaque thickness or total ICA plaque thickness, degree of ICA stenosis, ICA occlusion. Neither the ICA plaque thickness (correlation coefficient = 0.205, *P* value = 0.060) nor the total ICA plaque thickness (correlation coefficient = 0.158, *P* value = 0.149) was correlated with plasm Lp(a) concentration. Likewise, both of the degree of ICA stenosis (correlation coefficient = 0.118, *P* value = 0.281) and ICA occlusion (correlation coefficient = − 0.027, *P* value = 0.806) were also not correlated with Lp(a) concentration. After being divided into two groups based on Lp(a) concentration, there were similarly not association between the ICA plaque thickness, total ICA plaque thickness, degree of ICA stenosis, ICA occlusion and whether Lp(a) ≥ 300 mg/L or not.(Table 3).

**Table 3:** Association between Lp(a) and maximum ICA plaque thickness and total ICA maximum plaque thickness

	Lp(a) (mg/L)		Lp(a) (≥ 300 mg/L vs < 300 mg/L)	
	Correlation coefficient	*P* value	Correlation coefficient	*P* value
Maximum ICA plaque thickness (mm)	0.205	0.060	0.118	0.283
Total ICA maximum plaque thickness (mm)	0.158	0.149	0.097	0.379
Degree of ICA stenosis	0.118	0.281	0.022	0.845
ICA occlusion	− 0.027	0.806	− 0.158	0.149

Lp(a), Lipoprotein(a); ICA, Internal carotid artery

Table 4a shows that Lp(a), in the univariate analysis, was a significant predictor of IPN in carotid stenosis patients, with per 100 mg/L increasing associated with 1.238-fold higher hazard (95% CI 1.020, 1.503, *P* value = 0.031) of total IPN ≥ 2. After stepwise adjusting for covariates, per 100 mg/L increasing associated with 1.305-fold higher hazard (95% CI 1.045, 1.628, *P* value = 0.019) of total IPN ≥ 2.

**Table 4:** (a) Association between Lp(a) (per 100 mg/L) and IPN group, (b) Association between Lp(a) (≥ 300 mg/L vs < 300 mg/L) and IPN group

	OR (95% CI)	*P* value
(a)		
Unadjusted	1.238 (1.020, 1.503)	0.031
Model 1	1.333 (1.074, 1.655)	0.009
Model 2	1.321 (1.059, 1.648)	0.014
Model 3	1.305 (1.045, 1.628)	0.019
(b)		
Unadjusted	2.828 (1.055, 7.580)	0.039
Model 1	3.260 (1.174, 9.058)	0.023
Model 2	2.798 (0.975, 8.033)	0.056
Model 3	2.411 (0.838, 6.936)	0.103

Lp(a), Lipoprotein(a); IPN, intraplaque neovascularization; OR, odds ratio; CI, Confidence interval

Model 1: adjusted for age, sex, smoking, hypertension, diabetes mellitus, cerebral fraction, coronary heart disease and statin therapy

Model 2: adjusted for model 1 plus total cholesterol, triglyceride, low-density lipoprotein cholesterol calculated by Friedwald's formula, high-density lipoprotein cholesterol, apolipoprotein A and apolipoprotein B

Model 3: adjusted for model 1, model 2 plus maximum plaque thickness and total carotid maximum plaque thickness, degree of carotid stenosis and ICA occlusion

Lp(a) ≥ 300 mg/L group, in the unadjusted model, was associated with 2.828-fold higher hazard (95% CI 1.055, 7.580, *P* value = 0.039) of total IPN ≥ 2 vs Lp(a) < 300 mg/L. After stepwise adjusting for other lipid parameters (Model 2) and maximum plaque thickness, total carotid maximum plaque thickness, degree of carotid stenosis, ICA occlusion (Model 3), the association did not reach statistical difference. (Table 4b).

## Discussion

Our study showed an association between plasma Lp(a) concentrations and IPN of the carotid artery confirmed by CEUS in patients with carotid stenosis, independent of other factors, such as age, sex, comorbid conditions, other lipid parameters, plaque thickness and degree of ICA stenosis. A higher plasma Lp(a) concentration was found to be significantly related to a higher risk of IPN, while both the plaque thickness and severity of ICA stenosis were not found to be related in this regard.

Plaque instability and progression are largely related to extensive IPN, which adds plaque susceptibility to rupture or haemorrhage [16]. Lp(a) has been associated with carotid stenosis and plaque stability [5]. Our study found that plasma Lp(a) was linked to IPN grade, suggesting that a higher plasma Lp(a) concentration may accelerate IPN formation and affect plaque stability, leading to cardiovascular and cerebrovascular events [4, 17, 18].

Furthermore, Johri Amer et al. reported that in those patients with severe coronary lesions (whose coronary artery stenosis ≥ 70%), IPN grade of the carotid artery was associated with coronary lesion degree and complexity [19]. A recent study has demonstrated that carotid plaque neovascularization could predict significant and complex coronary artery disease (CAD) and future cardiovascular events after investigating carotid IPN in 459 stable angina patients referred for coronary angiography [4]. When those results are considered alongside the findings of this study, it seems that Lp(a) accelerates not only carotid IPN formation, but also coronary artery plaque, which corresponds with previous studies showing that plaque instability frequently co-exists at multiple vascular bed [16, 20, 21]. In our study, when we classified patients into IPN groups in terms of the CEUS grade on both sides of the carotid IPN added up to ≥ 2, we found that 83.8% of participants in the IPN group were affected on both sides, indicating that most of the patients had plaque instability at multiple sites.

Based on the high degree of homology between apo(a) and plasminogen [8], a number of researches found that some of apo(a) fragments played a role of anti-angiogenesis which was similar to the function of plasminogen[9, 10]. Nevertheless, the above results were from the small samples study in vitro, there is still lack of pathophysiological role of the anti-angiogenic or angiogenic activity in humans [22]. Meanwhile, the concentration of Lp(a) tested in most studies was far below the clinical risk threshold. Although Iwabayashi Masaaki and colleagues found the Lp(a) impaired the function of endothelial cells and endothelial progenitor cells from human aortic, leading to tubule formation inhibit, the concentration of Lp(a) treated in this experiment was only 5 mg/dL [23]. At the higher concentrations, Liu L et al. has demonstrated the effect on stimulation of migration and proliferation of human umbilical-vein endothelial cells [11]. In this recent paper, we detected IPN in carotid stenosis patients sensitively and invasively by use of CEUS and compared Lp(a) concentration between two groups, indirectly demonstrated that Lp(a) may potentially result in plaque stabilization by the mechanism of avoiding IPN formation. Also, further large randomized controlled studies are warranted to validate.

In addition, our results support that lower Lp(a) would be worthy of attention to prevent cardiovascular and cerebrovascular events, especially given that statins, the most used lipid-lowering drugs, cannot reduce Lp(a) levels. Proprotein convertase subtilisin/Kexin type 9 (PCSK9) inhibitors have been confirmed to lower Lp(a) and should be considered as an independent treatment after acute coronary syndrome [24, 25]. Clinical trials, however, have shown that Lp(a) levels have only been reduced by 20–30% [26–28]. Other traditional Lp(a)-lowering approaches, such as the use of niacin, mipomersen, lomitapide, and so on, have been showed that the limited and non-specific effect to lower Lp(a) with intolerable side effects, invasive procedures, and high expense [29]. However, the apo(a) ASO IONIS-Apo(a)-LRX has recently been shown to significantly reduce Lp(a) levels in phase 2 clinical trials with good tolerance [30]. There is ongoing phase 3 RCT trial [Lp(a)HORIZON, NCT04023552] and it may become a promising drug for the management of elevated Lp(a) in the future. To date, large scale randomized controlled trials have yet to be conducted to determine the precise cardiovascular benefits of lowering Lp(a) and further research is needed.

## Limitations

This study had several limitations. First, this was an observational study, with limited possibilities to draw causal inferences. Second, it was a single-centre study, consisting of only 85 patients with carotid stenosis, and all the patients were diagnosed and treated at Guangdong General Hospital. Therefore, our study findings cannot be readily generalized, and future studies with populations of different ethnicities and comprising multiple centres are recommended. Third, our study did not take genetic variants into consideration, although plasma Lp(a) levels are mainly determined by genetic factors while are not significantly reduced through lifestyle interventions. Therefore, further studies using genetic approaches are warranted. What’s more, while more than 90% of our participations were treated by statin regimen, LDL-C levels were above recommended thresholds that would contribute to IPN in the plaque which may bias the relationship between Lp(a) concentration and IPN grade. However, after adjusting other lipid parameters, the relationship between Lp(a) concentration and IPN grade still remained statistical significance. But for the small sample sizes, large cohort studies or LDL-C-matched cohort studies are also warranted. Another limitation is histological validation could not perform, whether the observed relationship is affected by inflammatory component of Lp(a). Sensitively determining IPN by CEUS, however, has been widely accepted [3, 4]. So, there is reason to believe the association observed in this study was mainly due to the effect on angiogenesis. Finally, follow-up data were not collected for cardiovascular and cerebrovascular disease events. However, the relationship between Lp(a) levels and prognosis with carotid stenosis deserves further study.

## Conclusions

Plasma Lp(a) concentrations were found to be independently associated with IPN in patients with carotid stenosis. As the concentration of Lp(a) increases, the risk of IPN increases. Lowering plasma Lp(a) levels may help to maintain plaque stability through slowing down IPN formation, as assessed using CEUS. Large prospective studies assessing the utility of Lp(a) to predict IPN in the clinical setting are required. Randomised clinical trials are needed to test whether substantial reductions in Lp(a) concentrations using the various treatments identified, most notably, the apo(a) ASO IONIS-Apo(a)-LRX, may facilitate improved management of individuals with high Lp(a) levels.

## Data Availability

The datasets generated and analysed during the current study are not publicly available due to the privacy protection and a number of researches on Lp(a) may be continued, but are available from the corresponding author (e-mail:gdghllw@163.com) on reasonable request. The processed data required to reproduce these findings cannot be shared at this time as the data also form part of an ongoing study.

## Abbreviations

Lp(a): Lipoprotein(a)
ASCVD: Arteriosclerotic cardiovascular disease
IPN: Intraplaque neovascularization
CEUS: Contrast-enhanced ultrasound
TC: Cholesterol
TG: Triglyceride
LDL-C: Low-density lipoprotein cholesterol
LDL: Low-density lipoprotein
apo: Apolipoprotein
OR: Odds ratios
CAD: Coronary artery disease
ICA: Internal carotid artery
